# Role of peripheral 5-HT_5A_ receptors in 5-HT-induced cardiac sympatho-inhibition in type 1 diabetic rats

**DOI:** 10.1038/s41598-020-76298-6

**Published:** 2020-11-09

**Authors:** José Ángel García-Pedraza, Oswaldo Hernández-Abreu, Asunción Morán, José Carretero, Mónica García-Domingo, Carlos M. Villalón

**Affiliations:** 1 Laboratory of Pharmacology, Department of Physiology and Pharmacology, Faculty of Pharmacy, University of Salamanca, Biomedical Research Institute of Salamanca (IBSAL), 37007 Salamanca, Spain; 2 Department of Pharmacobiology, Cinvestav-Coapa, Czda. Tenorios 235, Col. Granjas-Coapa, Deleg. Tlalpan, C.P. 14330 Mexico City, Mexico; 3 Laboratory of Neuroendocrinology, Department of Human Anatomy and Histology, Faculty of Medicine, University of Salamanca, Neurosciences Institute of Castilla y León (INCyL), Salamanca, Spain; 4 Laboratory of Neuroendocrinology and Obesity, IBSAL, Salamanca, Spain

**Keywords:** Molecular biology, Cardiovascular biology

## Abstract

5-HT inhibits cardiac sympathetic neurotransmission in normoglycaemic rats, via 5-HT_1B_, 5-HT_1D_ and 5-HT_5A_ receptor activation. Since type 1 diabetes impairs the cardiac sympathetic innervation leading to cardiopathies, this study aimed to investigate whether the serotonergic influence on cardiac noradrenergic control is altered in type 1 diabetic rats. Diabetes was induced in male Wistar rats by streptozotocin (50 mg/kg, i.p.). Four weeks later, the rats were anaesthetized, pithed and prepared for producing tachycardic responses by electrical preganglionic stimulation (C_7_-T_1_) of the cardioaccelerator sympathetic outflow or i.v. noradrenaline bolus injections. Immunohistochemistry was performed to study 5-HT_1B_, 5-HT_1D_ and 5-HT_5A_ receptor expression in the stellate ganglion from normoglycaemic and diabetic rats. In the diabetic group, i) i.v. continuous infusions of 5-HT induced a cardiac sympatho-inhibition that was mimicked by the 5-HT_1/5A_ agonist 5-carboxamidotryptamine (without modifying noradrenaline-induced tachycardia), but not by the agonists indorenate (5-HT_1A_), CP 93,129 (5-HT_1B_), PNU 142633 (5-HT_1D_), or LY344864 (5-HT_1F_); ii) SB 699551 (5-HT_5A_ antagonist; i.v.) completely reversed 5-CT-induced cardiac sympatho-inhibition; and iii) 5-HT_5A_ receptors were more expressed in the stellate ganglion compared to normoglycaemic rats. These results show the prominent role of the peripheral 5-HT_5A_ receptors prejunctionally inhibiting the cardiac sympathetic drive in type 1 diabetic rats.

## Introduction

The main causes of high morbidity and mortality in patients suffering from type 1 diabetes (T1D) continue to be cardiovascular complications, which are ten-fold increased in patients with T1D than in the general population^1,2^. Despite the notable improvement in life expectancy, patients with type 1 diabetes confront this increased cardiovascular risk, as it is silent in the at early stages of the disease (underestimated event), and due to the longer duration of the diabetic state compared to type 2 diabetes^3–5^. One of the triggers for cardiovascular disorders associated with diabetes is the sympathetic nervous system disorder at the cardiac level^6,7^. Indeed, an increase in sympathetic (i.e. noradrenergic) heart tone has been established to contribute to myocardial damage and therefore to cardiac disorders in patients with T1D^4,8–10^, as well as in animal models of T1D^11,12^. The impairment of cardiac sympathetic neurotransmission, among others, is strongly involved in the development of cardiac autonomic neuropathy (CAN), a microvascular complication leading to dysfunctional heart rhythm and vascular dynamics^4,9,10^. In view of all this, the down-regulation of cardiac noradrenergic neurotransmission may be a potential therapeutic approach to manage these cardiovascular disorders in T1D.

Within this context, 5-HT stands out as a neurohormone capable of regulating the sympathetic nervous system; certainly, the serotonergic system has been shown to modulate peripheral sympathetic neurotransmission at both the vascular and cardiac levels^13–19^. With respect to perivascular sympathetic innervation in pithed rats, our research team has already demonstrated that 5-HT induces sympatho-inhibition in the vasopressor discharge, mainly through activation of prejunctional 5-HT_1A_ and 5-HT_1D_ receptors^13,14^. However, the induction of T1D in rats altered the pharmacological profile of the vascular sympatho-inhibitory 5-HT receptors (which depended on the duration of diabetes). Specifically, our results revealed the exclusive role of prejunctional 5-HT_1A_ receptors in 28-day (short-term) diabetic rats^20^, and the additional role (besides 5-HT_1A_ receptors) of prejunctional 5-HT_2A_ receptors in 56-day (long-term) diabetic rats^21^. On the other hand, regarding cardiac sympathetic control, 5-HT decreases the sympathetic chronotropic outflow in normoglycaemic-pithed rats through prejunctional 5-HT_1B_, 5-HT_1D_ and 5-HT_5A_ receptors^15–17,22^. Despite the key participation of the serotonergic system in: (a) the pathophysiology of T1D where there are alterations in the central and peripheral serotonergic effects^23–27^; and (b) the control of peripheral sympathetic neurotransmission in normoglycaemic and diabetic states, no study has yet determined the specific role of the 5-HT system in cardiac sympathetic neurotransmission in T1D.

Based on the above findings, the objective of the present study was to examine the role of the 5-HT system in the cardiac sympathetic modulation during T1D. In particular, the impact of 28-day T1D in rats was evaluated on: (a) the tachycardic responses induced by cardio-selective sympathetic stimulation and i.v. bolus injections of exogenous noradrenaline; (b) 5-HT-induced inhibition of the sympathetically-induced tachycardic responses and the pharmacological profile of the 5-HT receptor (sub) types involved; and (c) the expression of different 5-HT receptors in the stellate (sympathetic) ganglion.

## Results

### Systemic haemodynamic parameters

As compared to the normoglycaemic (vehicle-injected) rats, streptozotocin (STZ) administration elicited a marked increase in blood glucose concentration (mg/dl) and a decrease in body weight (g). Table 1 shows the mean values of these parameters before (initial) and 4 weeks after the administration of STZ or its vehicle in rats.

**Table 1:** Monitored parameters in normoglycaemic and diabetic rats.

	Normoglycaemic		Diabetic	
	Initial	After 4 weeks	Initial	After 4 weeks
Blood glucose (mg/dl)	107 ± 2	106 ± 2	108 ± 1	548 ± 20*
Body weight (g)	205 ± 5	285 ± 10*	405 ± 3	294 ± 2*

Blood glucose (mg/dl) and body weight (g) in rats before (initial) and 4 weeks after the i.p. administration of vehicle (normoglycaemic group; n = 45) or STZ (diabetic group; n = 75). *P < 0.05 versus initial time (before administration of vehicle or STZ).

Certainly, at the very beginning of the experiments, we used two different body weights for the administration of vehicle or STZ because the tachycardic responses induced by electrical stimulation are optimally produced in animals weighing around 290 g^28,29^. Thereupon, as the i.p. administration of STZ resulted in a marked loss of body weight, we decided to use animals of approximately 400 g so that, after 4 weeks, this body weight decreased to approximately 290 g. In contrast, for the i.p. administration of vehicle we used animals of approximately 200 g in order that, after 4 weeks, this body weight increased to around 290 g (approximating that in diabetic rats; see Table 1).

Furthermore, the baseline values of heart rate and diastolic blood pressure in the normoglycaemic rats (n = 40) were 249 ± 7 beats/min and 50 ± 2 mmHg, respectively; whereas in the diabetic rats (n = 70) these values were 219 ± 7 beats/min and 48 ± 1 mmHg, respectively. When comparing these values between the normoglycaemic and diabetic rats, diastolic blood pressure values did not significantly differ (*P* > 0.05), but the heart rate values were significantly lower in diabetic rats (*P* < 0.05). After the first injection of desipramine, both variables transiently increased (*P* < 0.05) to: 266 ± 4 beats/min and 57 ± 1 mmHg in normoglycaemic rats, and 230 ± 12 beats/min and 58 ± 3 mmHg in diabetic rats. These values returned to baseline levels after 10 min. These cardiovascular parameters were not varied (*P* > 0.05) by the successive desipramine administrations. Additionally, the basal heart rate and basal diastolic blood pressure were not significantly changed (*P* > 0.05) by the i.v. continuous infusions of saline and of the 5-HT receptor agonists, or by the i.v. bolus injections of saline and of the 5-HT receptor antagonist in the distinct subgroups of desipramine-pretreated rats (not shown).

### Effects elicited by electrical stimulation of the cardioaccelerator noradrenergic outflow or i.v. bolus administrations of noradrenaline on the cardiovascular variables

The initiation of the tachycardia was instantaneous by both spinal stimulation (0.03–3 Hz) or i.v. bolus administrations of noradrenaline (0.03–3 μg/kg), resulting in frequency-dependent or dose-dependent increments in heart rhythm, respectively (Table 2). These tachycardic responses achieved a maximum around 40 s after ending the electrical stimulation or noradrenaline injections. Given that minor blood pressure variations were found, as reported before^15–17,22^, the electrically-induced tachycardic responses are due to specific stimulation of cardiac sympathetic innervation. Unlike sympathetic cardiac stimulation, exogenous noradrenaline elicited dose-dependent increases in diastolic blood pressure (not shown), as previously demonstrated^15–17,22^; these vasoconstrictions were not assessed further. In all situations, the increments in heart rate generated by both cardiac noradrenergic outflow and exogenous noradrenaline were significant (*P* < 0.05) when compared with their corresponding baseline values.

**Table 2:** Tachycardic responses induced by cardiac sympathetic stimulation and exogenous noradrenaline.

	0.03	0.1	0.3	1	3	
	**Cardiac sympathetic electrical stimulation (Hz)**					∆ Heart rate (beats/min)
Normoglycaemic	14.1 ± 1.3	19.3 ± 1.8	31.6 ± 2.1	57.6 ± 2.2	76.8 ± 2.0	
Diabetic	14.3 ± 2.1	18.5 ± 2.3	29.7 ± 2.8	56.8 ± 3.1	74.8 ± 3.0	
	**Exogenous noradrenaline (µg/kg; i.v.)**					
Normoglycaemic	8.5 ± 1.1	27.6 ± 1.9	49.6 ± 4.0	68.9 ± 4.6	78.3 ± 6.0	
Diabetic	6.8 ± 1.0	13.5 ± 1.6*	20.6 ± 2.6*	60.5 ± 3.9	74.9 ± 3.1	

Comparative tachycardic effects induced by selective electrical stimulation of the cardioaccelerator sympathetic nerves (0.03–3 Hz) and exogenous noradrenaline (0.03–3 µg/kg; i.v.) in both normoglycaemic (n = 40) and diabetic (n = 70) pithed rats. *P < 0.05 versus normoglycaemic group. ∆ Heart rate, increase in heart rate.

Interestingly, the tachycardic responses induced by 0.1 and 0.3 µg/kg of exogenous noradrenaline in the diabetic group were significantly lower as compared with those in the control normoglycaemic group (Table 2). In contrast, there were no statistically significant differences between the S-R curves in both groups of rats (Table 2).

### Effect of i.v. continuous infusions of saline, 5-HT and related agonists on the tachycardic noradrenergic responses in normoglycaemic and diabetic rats

Just as previously reported by our group in normoglycaemic pithed rats anaesthetized with ether^15–17^, in normoglycaemic pithed rats anaesthetized with pentobarbital and pretreated i.p. with STZ vehicle (present experimental conditions), the i.v. continuous infusions of: (i) 5-HT (3 and 10 μg/kg min), 5-CT (0.1 μg/kg min), CP 93,129 (30 μg/kg min) or PNU 142633 (30 μg/kg min) inhibited the sympathetic tachycardic responses (data not shown); and (ii) saline (0.02 ml/min), indorenate (30 μg/kg min) or LY344864 (30 μg/kg min) were inactive (data not shown). Thus, the above infusion doses of compounds were chosen for further analysis in diabetic animals.

**Figure 1:**
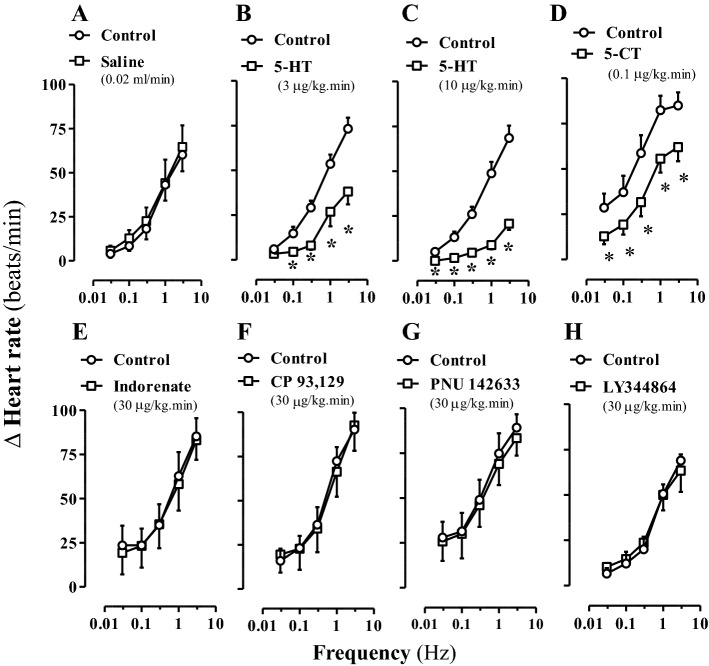
Effect of 5-HT receptor agonists on the sympathetic tachycardic responses in diabetic pithed rats. Tachycardic responses produced by cardiac sympathetic stimulation before (control) and during i.v. continuous infusions of: saline (**A**); 5-HT (**B**, **C**); 5-CT (**D**); indorenate (**E**); CP 93,129 (**F**); PNU 142633 (**G**); or LY344864 (**H**) (n = 5 each subgroup; n = 40). *P < 0.05 versus control. Δ Heart rate stands for “increase in heart rate”.

Remarkably, as shown in Fig. 1, in diabetic rats only the continuous infusions of 5-HT (B and C) and 5-CT (D) inhibited (*P* < 0.05) the sympathetically-induced tachycardic responses, while those of saline (A), indorenate (E), CP 93,129 (F), PNU 142633 (G), and LY344864 (H) were inactive (Fig. 1). The percentage of sympatho-inhibition induced by 5-CT (0.1 μg/kg min; i.v.) in diabetic rats was 55.2%, 48.0%, 46.0%, 32.4% and 27.1% at 0.03, 0.1, 0.3, 1 and 3 Hz, respectively; this degree of sympatho-inhibition was similar (*P* > 0.05) to that found in normoglycaemic animals, showing a slight increase at 0.1, 0.3 and 1 Hz, as reported earlier^22^.

**Figure 2:**
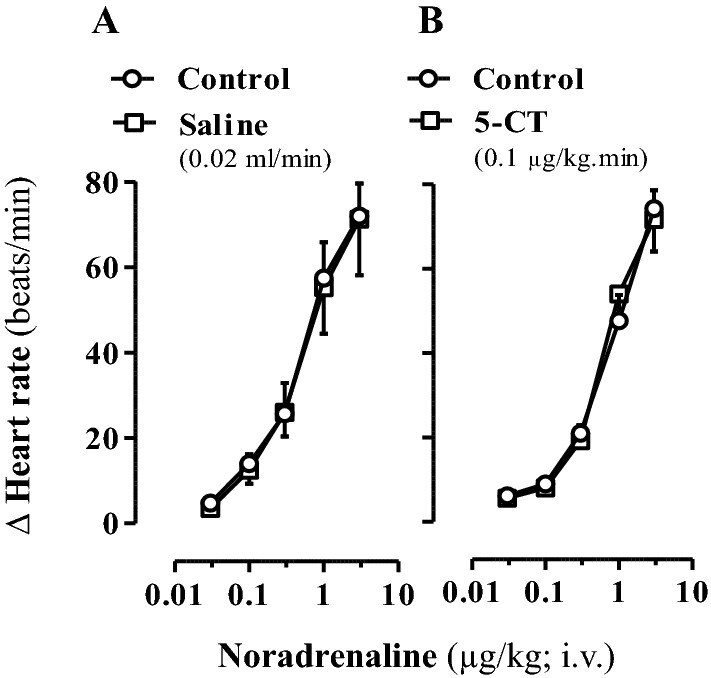
Effect of 5-CT on the noradrenaline-induced tachycardic responses in diabetic pithed rats. Tachycardic responses to i.v. bolus injections of exogenous noradrenaline before (control responses) and during i.v. continuous infusions of saline (**A**) or 5-CT (**B**) (n = 5 each group; n = 10). Note that non-significant effects (*P* > 0.05) were produced by the i.v. infusions of saline or 5-CT. Δ Heart rate stands for “increase in heart rate”.

In view that, in contrast with control normoglycaemic rats (see above), 5-CT was the only agonist mimicking the 5-HT-induced cardiac sympatho-inhibion (Fig. 1D), Fig. 2 further shows that neither saline (0.02 ml/min; A) nor 5-CT (0.1 μg/kg min; B) affected (*P* > 0.05) the tachycardic responses to exogenous noradrenaline in diabetic rats.

### Effect of SB 699551 on the cardiac sympatho-inhibition produced by 5-CT in diabetic rats

**Figure 3:**
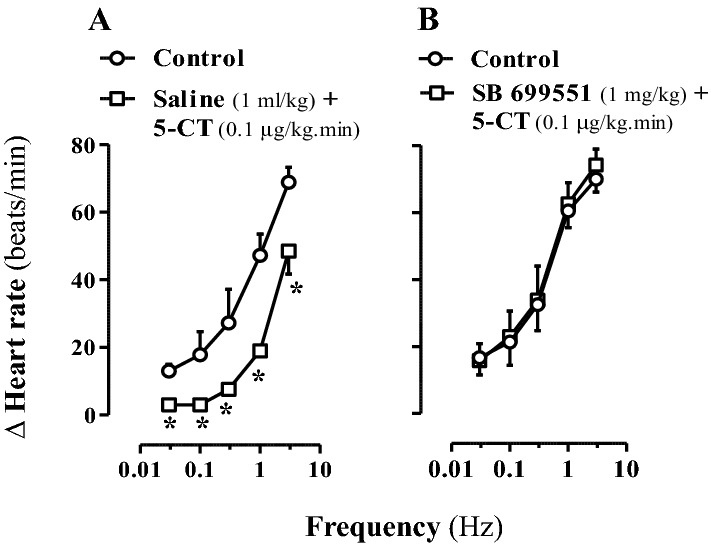
Effect of saline or SB 699551 on 5-CT-induced sympatho-inhibition in diabetic pithed rats. Effect of i.v. bolus injections of saline (**A**) or SB 699551 (**B**) on the 5-CT-induced inhibition of tachycardic responses evoked by cardiac sympathetic stimulation (n = 5 each subgroup; n = 10). *P < 0.05 versus control. Δ Heart rate stands for “increase in heart rate”.

Figure 3 illustrates the effect of i.v. injections (n = 5 each) of saline (1 ml/kg; A) or the 5-HT_5A_ receptor antagonist SB 699551 (1 mg/kg; B) on the cardiac sympatho-inhibition to 5-CT (0.1 μg/kg min). Interestingly, the sympatho-inhibition to 5-CT, remaining unaffected after 1 ml/kg saline (Fig. 3A), was abolished after 1 mg/kg SB 699551 (Fig. 3B), a dose that completely blocks rat 5-HT_5A_ receptors^22,30^. The above i.v. doses of saline or SB 699551 were devoid of any effect per se (*P* > 0.05) on the sympathetically-induced tachycardic responses or on baseline heart rate and diastolic blood pressure (data not shown).

### Expression of the 5-HT_1B_, 5-HT_1D_ and 5-HT_5A_ receptor subtypes in the stellate ganglion of normoglycaemic and diabetic rats

**Figure 4:**
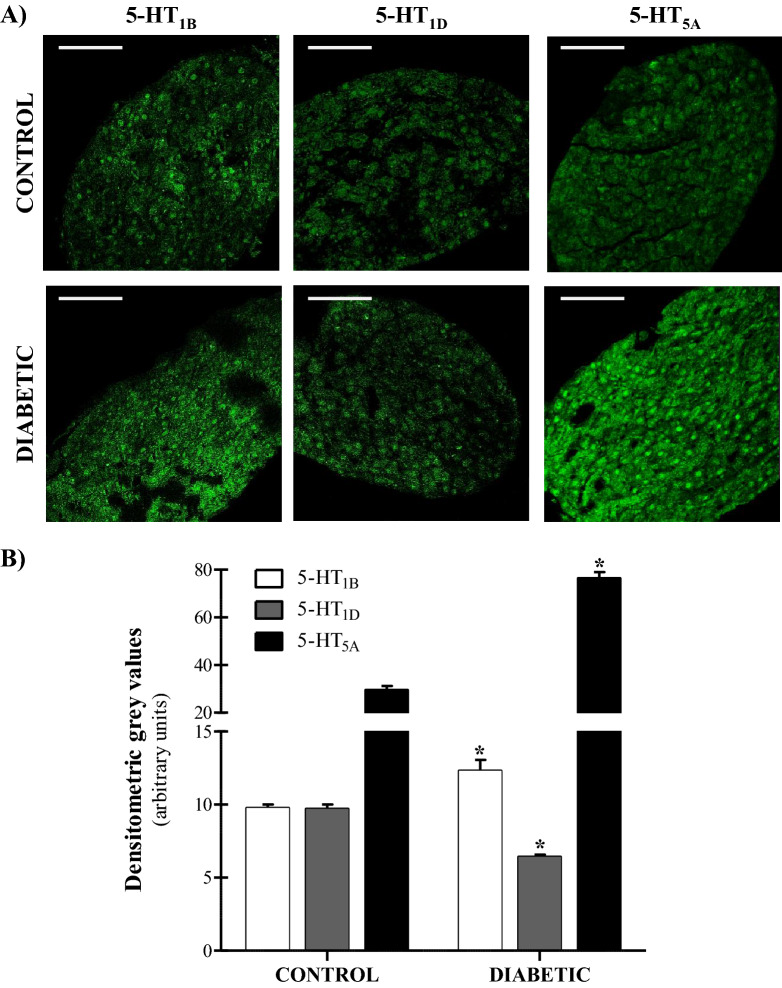
Immunohistochemical analysis of 5-HT receptors expression in the stellate ganglion of normoglycaemic and diabetic rats. Study of the immunopositive reaction for 5-HT_1B_, 5-HT_1D_ and 5-HT_5A_ receptors in the cells of stellate ganglion obtained from normoglycaemic (n = 5) and diabetic (n = 5) rats. (**A**) Photomicrographs showing the green-coloured reaction of the immunopositive-cells in stellate ganglion for 5-HT receptors in normoglycaemic and diabetic rats (white horizontal scale bar: 50 µm). ImageJ 1.49 free software (by the National Institutes of Health, U.S.A; https://imagej.nih.gov/ij). (**B**) Plot showing the densitometric grey intensities of immunopositive reactions to 5-HT receptors in normoglycaemic and diabetic rats. The plot values are expressed as the mean ± SEM. *P < 0.05 versus normoglycaemic values.

As presented in Fig. 4, the stellate ganglion cells show immunopositive-cells for the 5-HT_1B_, 5-HT_1D_ and 5-HT_5A_ receptor subtypes (Fig. 4A; white horizontal scale bar: 50 µm). Regardless of the subtype of 5-HT receptor analysed, the reaction was located in the nuclei and cytoplasm of immunopositive-cells.

In normoglycaemic (control) animals (Fig. 4B), the 5-HT_5A_ receptor showed a higher intensity of reaction (fluorescence), followed by the 5-HT_1B_ and then by the 5-HT_1D_ receptor. Interestingly, experimental T1D caused a striking and significant increase in the fluorescence intensity of the 5-HT_5A_ receptor, and less marked (but statistically significant) in the 5-HT_1B_ receptor as compared to normoglycaemic rats. On the contrary, the fluorescence intensity of the 5-HT_1D_ receptor was slightly -but significantly- reduced in animals with T1D when compared with control animals (Fig. 4B).

## Discussion

This study is the first of its kind to establish the prominent relevance of peripheral 5-HT_5A_ receptors modulating the functionality of cardiac sympathetic innervation in a rat model of T1D. To achieve this, we used the widely known model of T1D induced by STZ administration in rats. This diabetogenic agent causes a syndrome resembling human T1D, characterized by chronic and sustained hyperglycaemia, polyuria, polydipsia, and weight loss, among others, leading to the probable development of both micro and macroangiopathies related to diabetes^31,32^.

In addition to the possible therapeutic repercussions derived from this study, the main –and novel- findings of the present investigation in rats with T1D reveal that: (i) 5-HT maintains its cardiac sympatho-inhibitory effects, which were exclusively and potently reproduced by 5-CT (a 5-HT_1/5A_ receptor agonist^16^); (ii) from a pharmacological perspective, prejunctional 5-HT_5A_ receptors were the only functional receptors involved in the serotonergic cardiac sympatho-inhibition, in view of the complete reversion of 5-CT-induced sympatho-inhibition by the selective 5-HT_5A_ receptor antagonist SB 699551 (see below); and (c) in agreement with the latter finding, 5-HT_5A_ receptors were more expressed in the stellate (sympathetic) ganglion from diabetic rats.

Admittedly, the noradrenaline release resulting from stimulation of the cardioaccelerator sympathetic nerves was assessed indirectly by measuring the elicited tachycardic responses, as previously described^15^. Under these conditions, the responses to 5-CT in diabetic rats were considered sympatho-inhibitory (i.e. prejunctional in nature) as this 5-HT_1/5A_ receptor agonist inhibited the tachycardic responses to cardiac sympatho-stimulation without affecting those to exogenous noradrenaline. In fact, our group has already shown in normoglycaemic rats that 5-HT, 5-CT, CP 93,129 and PNU 142633 produce a dose-dependent prejunctional inhibition of the cardiac sympathetic neurotransmission, i.e. without affecting the tachycardic responses to exogenous noradrenaline^15–17,22^.

Since pithed rats were used in this study, any influence of the central nervous system (which can produce baroreflex compensatory mechanisms in response to the effects produced by 5-HT and related agonists) can be excluded. Consequently, the nature of this experimental model helps explain why its resting values for both diastolic blood pressure and heart rate are much lower than those measured in anaesthetised (non-pithed) rats, where the neurogenic sympathetic tone is active^18,19,24,33^.

Considering the above, the baseline values of diastolic blood pressure in diabetic rats (48 ± 1 mmHg) were not significantly different from those obtained in normoglycaemic rats (50 ± 2 mmHg). This suggests that the systemic vascular tone and, therefore, the functionality of the systemic vasculature in diabetic rats (in the early stage of T1D) appears to be unaffected by 28-day T1D, as previously described^28,29^. In contrast, baseline heart rate values in diabetic rats were significantly lower (219 ± 7 beats/min) than those determined in normoglycaemic rats (249 ± 7 beats/min). This finding coincides with our results previously found in both pithed and conscious rats, with a bradycardic effect that prevails in type 1 diabetic animals^28,29^. These lower heart rate values in diabetic rats may be related to altered heart electrophysiological properties and/or a dysfunction of cardiac β-adrenergic receptors during the diabetic state^34,35^.

Notwithstanding this possible cardiac abnormality in diabetic rats, the release of noradrenaline after cardio-selective sympathetic stimulation produced increases in heart rate that did not significantly differ from those in normoglycaemic animals (see Table 2); consequently, the tachycardic responses after electrical stimulation appear to be unaffected in T1D. As noted above, we did not measure the release of noradrenaline after cardio-sympathetic stimulation in both groups. This approach would clearly require other experimental designs that are beyond the scope of the present study. However, tachycardic responses evoked by exogenous noradrenaline in diabetic rats were slightly lower (although only statistically significant at 0.1 and 0.3 µg/kg of noradrenaline) than those found in normoglycaemic rats (see Table 2). On this basis, it could be speculated that: (a) the proven impairment of the cardiac β-adrenergic system in T1D may be due to the attenuation of the tachycardic responses to exogenous noradrenaline and the decrease in baseline heart rate values; and (b) despite this cardiac damage, the release of endogenous noradrenaline from cardiac sympathetic nerves could be increased, as already shown in the experimental models of T1D, which involve an increase in both the release and storage of catecholamines^36^. The last finding would help us to explain why the tachycardia induced by the stimulation of cardioaccelerator nerves in diabetic animals did not significantly differ from that obtained in normoglycaemic animals.

The mechanism of action of desipramine (noradrenaline-reuptake inhibitor) justifies that heart rate and diastolic blood pressure values were briefly enhanced after its administration in both animal groups, as described before^15^. This augmentation of electrically-induced tachycardia^15^ is relevant in our study, since the sympatho-inhibitory effect evoked by 5-HT (and by other monoamines) and serotonergic agonists is more marked at lower frequencies of electrical stimulation^37^.

On the other hand, that the tachycardic responses produced by electrical stimulation or exogenous noradrenaline in diabetic rats remained unaffected by saline (given as i.v. continuous infusions or i.v. injections) implies that these responses are highly reproducible, as previously reported^15–17,22^. Thereby, the proposed sympatho-inhibition by 5-HT and 5-CT is not due to a tachyphylaxis phenomenon of the tachycardic responses, and no time-dependent variations occurred during the course of our experimental protocols. Furthermore, since 1 mg/kg of the 5-HT_5A_ receptor antagonist SB 699551 did not affect per se the sympathetic tachycardic responses or the baseline haemodynamic variables (see Results section), this dose was considered appropriate to identify (through a direct interaction) the 5-HT receptor involved in 5-CT-induced sympatho-inhibition (see below), as demonstrated in normoglycaemic rats^22,30^.

It must be emphasised that the dose used of SB 699551 was high enough to block completely and selectively 5-HT_5A_ receptors in our study, based on two main findings, namely: (i) its affinity profile, with a pK_i_ = 8.2 for 5-HT_5A_ receptors, a pK_i_ ≤ 6.5 for 5-HT_1A_, 5-HT_1B_ and 5-HT_1D_ receptors, and a pK_i_ < 5 for 5-ht_1E_ and 5-HT_1F_ receptors^38^; and (ii) its potency to block functional cardiovascular (5-CT-induced sympatho-inhibition) responses in normoglycemic pithed Wistar rats^22,30^.

On this basis, the fact that SB 699551 clearly abolished 5-CT-induced cardiac sympatho-inhibition in type 1 diabetic rats (Fig. 3B) raises the involvement of prejunctional 5-HT_5A_ receptors, with little or no pharmacological possibility to interact with 5-HT_1A/1B/1D/1E/1F_ receptors. This suggestion is further reinforced by considering three additional findings, namely:

(i) 0.1 µg/kg min of 5-CT, an agonist with high affinity for 5-HT_1A/1B/1D/1F_ and 5-HT_5A_ receptors^22^, inhibited the tachycardic responses evoked by sympathetic nerve stimulation (Fig. 1D) without affecting those by exogenous noradrenaline (Fig. 2B).
(ii) The agonists indorenate (5-HT_1A_), CP 93,129 (5-HT_1B_), PNU 142633 (5-HT_1D_), or LY344864 (5-HT_1F_)^22^ significantly inhibited the sympathetic tachycardic responses (but not those by exogenous noradrenaline) in normoglycaemic pithed rats^16,17,22^. However, the same infusion doses of the above agonists failed to inhibit these sympathetic tachycardic responses in type 1 diabetic rats (Fig. 1E–H).
(iii) 5-HT_5A_ receptors (like 5-HT_1_ receptors) are coupled to heterotrimeric G_i/o_ proteins that, amongst other effects, inhibit adenylyl cyclase activity, and this is associated with prejunctional inhibition of the neuronal release of noradrenaline and other neurotransmitters^39,40^.

Admittedly, 5-CT also shows affinity for 5-HT_7_ receptors^41^, but the involvement of these receptors in the present study can be ruled out for a number of reasons, which include that: (i) 5-HT-induced cardiac sympatho-inhibition in normoglycaemic rats, which is resistant to blockade by LY215840 (a selective 5-HT_7_ receptor antagonist), was abolished by selective antagonists at 5-HT_1B_, 5-HT_1D_ and 5-HT_5A_ receptors^16,17,22^; and (ii) 5-CT-induced cardiac sympatho-inhibition in diabetic rats was completely blocked by SB 699551 (a selective 5-HT_5A_ receptor antagonist; current results).

Surprisingly, in our experimental model of T1D: 5-HT maintained its capability to inhibit dose-dependently the cardiac sympathetic outflow (Fig. 1B, C), just as shown in normoglycaemic rats^15,16^ (present study); and 5-CT apparently produced a greater percentage of cardiac sympatho-inhibition (see Results section). Overall, these effects, predominantly mediated by activation of prejunctional 5-HT_5A_ receptors (as suggested above), imply a change in the modulation by 5-HT of heart sympathetic neurotransmission in type 1 diabetic rats. This suggestion is reinforced by previous findings in type 1 diabetic rats that establish a change in the modulation by 5-HT receptors of the perivascular sympathetic neurotransmission^14,20^ and the heart parasympathetic neurotransmission^42,43^.

The above findings, as a whole, further allow us to hypothesize that the cardiovascular damage caused by chronic hyperglycaemia, primarily evidenced at the cardiac level (as discussed above), may cause the cardiac sympatho-inhibitory 5-HT_1B/1D_ receptors to “lose” somehow their functionality. This, in turn, would result in a higher presence of 5-HT_5A_ receptors as a compensatory mechanism to modulate the cardiac sympathetic neurotransmission in the diabetic state. Similarly, other studies have associated the cardiovascular damage and endothelial dysfunction present in diabetes with peripheral serotonergic alterations^20,21,24,43–45^, even changing other peripheral systems related with α-adrenergic, dopaminergic and histaminergic mechanisms^28,29^.

To further strengthen our pharmacological findings and the above hypothesis posing the greater expression of 5-HT_5A_ receptors, we decided to examine the peripheral expression of the sympatho-inhibitory 5-HT receptors involved in both normoglycaemic and diabetic rats by immunohistochemistry. Within this context, the stellate ganglion is a key source of cardiac sympathetic innervation, connecting with multiple intrathoracic nerves and sympathetic structures where postganglionic fibers project to the heart^46^. Hence, we selected the stellate ganglion as one of the most suitable peripheral sympathetic structures to study the presence of the 5-HT receptors influencing the sympathetic discharge to the heart, namely, the 5-HT_1B_, 5-HT_1D_ and 5-HT_5A_ receptors. Our immunohistochemistry results reveal that the three receptor subtypes were expressed in both groups of animals, but the degree of expression was substantially different in diabetic animals (Fig. 4). Regarding the 5-HT_1_ receptor subtypes, the 5-HT_1D_ receptor decreased significantly in diabetic animals; this finding agrees with our current pharmacological results where none of the 5-HT_1_ receptors inhibited the tachycardic responses (Fig. 1E–H). In addition, a slight -but significant- increase in the expression of 5-HT_1B_ receptors was found, which could be related to a compensatory mechanism due to the decrease in 5-HT_1D_ receptor expression, but without functional relevance (i.e. with no noticeable sympatho-inhibition produced by the 5-HT_1B_ receptor agonist; Fig. 1F). Indeed, in direct relation to this view, other studies have already confirmed the existence of compensatory mechanisms in the serotonergic system of diabetic rats^47^. Nonetheless, the analysis of the 5-HT_5A_ receptor revealed a striking high presence in diabetic animals (Fig. 4), which may be associated with the functional control that this receptor subtype plays in the 5-HT-induced cardiac sympatho-inhibition in type 1 diabetic rats. Moreover, this study is the first of its kind confirming the expression of sympatho-inhibitory 5-HT receptors in a peripheral sympathetic ganglion in both control (normoglycaemic) and type 1 diabetic rats. In agreement with the findings that demonstrate the peripheral presence of 5-HT_5A_ receptors, some authors have also analyzed their expression in peripheral tissues and structures with an important role in cardiovascular regulation^48,49^. However, the vast majority of studies have linked 5-HT_5A_ receptors with serotonergic effects within the central nervous system^50^.

As mentioned in the Introduction section, among the cardiovascular abnormalities derived from T1D, CAN highlights with an estimated prevalence of 61.8% in type 1 diabetic patients^9,51^. The genesis of CAN is a combination of microvascular damage of the vasa nervorum and neuronal oxidative stress related to an augmented cardiac sympathetic tone, resulting in a sympatho-vagal imbalance and consequent tachycardia, decreased heart rate variability, exercise intolerance, dysrhythmias, silent myocardial impairment or sudden cardiorespiratory death in T1D^4,9,10^. Apart from this neuropathy, other diabetes-associated cardiac pathologies have been related to a sympathetic hyperinnervation, such as atrial fibrillation, congestive heart failure, or ventricular dysfunction^52–54^.

Obviously, the alteration of the serotonergic system plays an important role in the diabetes-derived complications^23–27^, and sympathetic denervation has shown benefits in pathologies related to sympathetic hyperactivity; however, this technique is not exempt from adverse effects^55^. With these lines of evidence in mind, our present findings further support the contention that cardiac sympatho-inhibitory 5-HT_5A_ receptors could represent a novel therapeutic strategy targeting certain cardiac disorders associated with T1D. Consistent with these results, dapagliflozin can induce a reduction in sympathetic activity that contributes to the renal- and cardio-protective effects of this drug for the treatment of diabetes^56^. Admittedly, further in vivo studies will be necessary to explore whether selective activation of 5-HT_5A_ receptors is effective in the treatment of CAN or other cardiac disturbances present in T1D.

On the other hand, some limitations of the present study should be considered when interpreting the current conclusions. We did not measure the expression of 5-HT_1B_, 5-HT_1D_ and 5-HT_5A_ receptors in the central nervous system; however, these receptors, unlike 5-HT_1A_ and 5-HT_2A_ receptors, do not seem to play important roles in the central serotonergic modulation of the cardiovascular responses^57^. Moreover, other biomarkers were not examined, which are characteristic of cardiac sympathetic nerve activity^58^, such as circulating catecholamine levels, cardiac beta adrenergic receptor expression, cardiac sympathetic nerve levels of important kinases in the cardiac function as G-protein-coupled receptor kinase 2 (GRK2)^58^, or even heart noradrenaline release (although it was indirectly measured by the electrically-induced tachycardia), since these molecular/biochemical approaches fall beyond the scope of the present study.

In conclusion, our study suggests that short-term T1D in rats modifies the pharmacological profile of the 5-HT receptors mediating cardiac sympatho-inhibition, so that prejunctional 5-HT_5A_ receptors appear to be the only receptor subtype involved in 5-HT-induced cardiac sympatho-inhibition. Consistent with this suggestion, peripheral 5-HT_5A_ receptors are remarkably more expressed in type 1 diabetic rats as compared to normoglycaemic rats. These findings provide new evidence that modulation of the peripheral 5-HT system in T1D may help control some cardiac disorders related to sympathetic dysregulation.

## Methods

### Animals and general methods

A total of 120 male Wistar rats (250–300 g) was used. The animals were maintained at a 12/12-h light/dark cycle (light beginning at 7 a.m.) and housed in a special room at constant temperature (22 ± 2 °C) and humidity (50%), with food and water ad libitum.

The rats (n = 120) were initially divided into 2 main sets, namely, set 1 (diabetic rats, n = 75) and set 2 (normoglycaemic rats, n = 45). In set 1, diabetes was induced by an i.p. injection of STZ (50 mg/kg) which was dissolved in citrate buffer (pH = 4.5); whereas set 2 received an i.p. injection of vehicle (citrate buffer, 1 ml/kg) as previously reported^28,29^. Both sets were kept for 4 weeks. Blood glucose levels and body weight were determined before and weekly after administration of STZ or vehicle. It is noteworthy that in set 1 all rats were considered for experimentation as they displayed elevated blood glucose levels (i.e. > 300 mg/dl) at all time-points. In all animals, blood glucose levels were measured using Accu-Chek strips and a glucometer (Accu-Chek Performa, Roche Diagnostics, Mannheim, Germany); and the body weight was measured using a triple beam granatary balance (Animal scale, Ohaus, Brandford, MA, U.S.A.).

**Figure 5:**
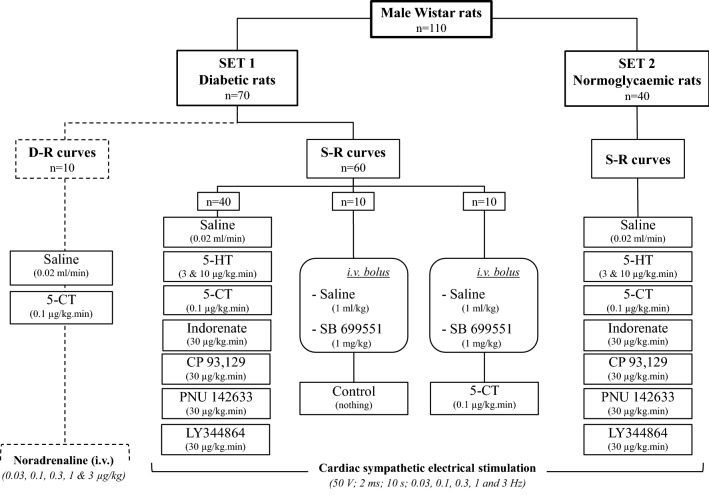
Experimental groups in the pharmacological study in pithed rats. Number of pithed rats used in the two main sets (set 1: diabetic rats; set 2: normoglycaemic rats) as well as their divisions into further treatments (n = 5 each, with no exception). The cardioaccelerator responses were obtained by either electrical sympathetic stimulation (set 1 and set 2, stimulus–response curves [S-R curves]; n = 100) or i.v. bolus injections of exogenous noradrenaline (set 1, dose–response curves [D-R curves]; n = 10).

After 4 weeks of treatment with STZ or vehicle, all animals were initially anaesthetized with sodium pentobarbital (60 mg/kg, i.p.). The adequacy of anaesthesia was judged by the absence of ocular reflexes, a negative tail flick test and corporal relaxation, amongst others. At this point, 5 normoglycaemic and 5 diabetic rats were used for analysing the expression of 5-HT receptors by immunohistochemistry (see section Immunohistochemistry analysis of 5-HT receptors). The rest of the rats (n = 110), consisting of set 1 (n = 70) and set 2 (n = 40), were used for pharmacological tests in pithed animals (see Fig. 5).

After cannulation of the trachea, the rats were pithed by inserting a stainless-steel rod through the ocular orbit and foramen magnum into the vertebral foramen. Immediately afterwards, the animals were artificially ventilated with room air using an Ugo Basile pump (56 strokes/min and a stroke volume of 20 ml/kg; Ugo Basile Srl, Comerio, VA, Italy)^22,29^. After cervical bilateral vagotomy, catheters were placed in: (i) the left and right femoral veins for the continuous infusions of agonists (or vehicle), and bolus injections of gallamine/desipramine or antagonists, respectively; and (ii) the left carotid artery, connected to a Grass pressure transducer (P23XL, Grass Instrument Co., Quincy, MA, U.S.A.) for the recording of blood pressure. Heart rate was measured with a tachograph (7P4F, Grass Instrument Co., Quincy, MA, U.S.A.) triggered from the blood pressure signal. Both blood pressure and heart rate were recorded simultaneously by a model 7D Grass polygraph (Grass Instrument Co., Quincy, MA, U.S.A.). Then, the responses to i.v. continuous infusions of vehicle (physiological saline) or the 5-HT receptor agonists were investigated on the tachycardic responses induced by: (i) selective preganglionic spinal (C_7_-T_1_) electrical stimulation of the cardioaccelerator sympathetic neurotransmission (n = 100, i.e. 60 rats from set 1 and 40 rats from set 2); or (ii) i.v. bolus injections of exogenous noradrenaline (n = 10 rats from set 1).

The tachycardic stimulus–response (S-R) curves and the dose–response (D-R) curves elicited by, respectively, preganglionic sympathetic stimulation and exogenous noradrenaline were completed in about 30 min, with no changes in the baseline values of resting heart rate or blood pressure. The sympathetic tachycardic stimulation (0.03–3 Hz) and the i.v. dosing with exogenous noradrenaline (0.03–3 μg/kg) were given using a sequential schedule in 0.5 log unit increments, at intervals of 5 min. The body temperature of each pithed rat was maintained at 37 °C by a lamp and monitored with a rectal thermometer.

### Experimental procedures

After a stable haemodynamic condition for at least 30 min, baseline values of heart rate and diastolic blood pressure (a more accurate indicator of peripheral vascular resistance) were determined. Then, the two sets of rats underwent the following experimental protocols (Fig. 5).

#### Protocol I. Electrical stimulation of the cardiac sympathetic innervation

In this protocol (n = 100, i.e. 60 from set 1 and 40 from set 2; see Fig. 5), the pithing rod was replaced by an electrode enamelled except for 1 cm length 7 cm from the tip, so that the uncovered segment was situated at C_7_-T_1_ of the spinal cord. This procedure allowed selective preganglionic stimulation of the cardioaccelerator sympathetic outflow^15^. Before electrical stimulation, the animals received gallamine (25 mg/kg, i.v.) to avoid electrically-induced muscular twitching. Since the cardiac sympatho-inhibitory responses to several agonists in pithed rats are particularly more pronounced at lower stimulation frequencies, all the animals were i.v. pretreated with 50 μg/kg of desipramine (a noradrenaline-reuptake inhibitor) 10 min before each S-R curve. This dose schedule of desipramine enhanced the tachycardic responses to sympathetic stimulation at low stimulation frequencies (without wearing off during the experiment) when compared to those in animals without desipramine^15^. Then, the cardioaccelerator sympathetic outflow was stimulated by applying trains of 10 s, consisting of monophasic rectangular pulses of 2 ms duration and 50 V, at increasing frequencies of stimulation (0.03, 0.1, 0.3, 1 and 3 Hz). When heart rate had returned to baseline levels, the next frequency was applied; this procedure was systematically performed until the S-R curve was completed. Subsequently, this set of animals was divided into two clusters (normoglycaemic and diabetic rats; see Fig. 5).

The first cluster (normoglycaemic; n = 40) was used here for confirming previous results from our group (using ether as an anaesthetic)^16,17^ under our current experimental conditions (i.e. anaesthesia with pentobarbital and i.p. administration of 1 ml/kg of STZ vehicle). This cluster was divided into 8 groups (n = 5 each) that received i.v. continuous infusions of: (i) saline (control, 0.02 ml/min); (ii) 5-HT (3 μg/kg min); (iii) 5-HT (10 μg/kg min); (iv) 5-CT (5-HT_1_/5-HT_5A_ receptor agonist, 0.1 μg/kg min); (v) indorenate (5-HT_1A_ receptor agonist, 30 μg/kg min); (vi) CP 93,129 (rodent 5-HT_1B_ receptor agonist, 30 μg/kg min); (vii) PNU 142633 (5-HT_1D_ receptor agonist, 30 μg/kg min); and (viii) LY344864 (5-HT_1F_ receptor agonist; 30 μg/kg min). Ten min after starting each infusion, a S-R curve was elicited again during the infusion of the corresponding compound.

The second cluster (diabetic; n = 60) was divided into 3 groups (n = 40, 10 and 10). The first group (n = 40), subdivided into 8 subgroups (n = 5 each), received i.v. continuous infusions of saline or the 5-HT receptor agonists exactly as described above for the first cluster (see Fig. 5). Ten min after starting each infusion, a S-R curve was elicited again as indicated before. The second group (n = 10), subdivided into 2 subgroups (n = 5 each), received i.v. injections of saline (1 ml/kg), or SB 699551 (5-HT_5A_ receptor antagonist, 1 mg/kg). Ten min later, a S-R curve was elicited again to analyse the effects of these compounds per se on the sympathetic tachycardic responses. And the third group (n = 10), subdivided into 2 subgroups (n = 5 each), received i.v. bolus injections of saline (1 ml/kg), or SB 699551 (1 mg/kg). Ten min later, these subgroups received an i.v. continuous infusion of 5-CT (0.1 μg/kg min). After 10 min, a S-R curve was elicited again as previously pointed out.

#### Protocol II. Intravenous bolus injections of exogenous noradrenaline

The last set of diabetic rats (n = 10) was prepared as described above, but the pithing rod was left and the administration of both gallamine and desipramine was omitted. Then, the rats received i.v. bolus injections of exogenous noradrenaline (0.03, 0.1, 0.3, 1 and 3 μg/kg) in order to induce dose-dependent tachycardic responses (*i.e.* a D-R curve), as previously reported^15–17,22^. Subsequently, this set, divided into two groups (n = 5 each), received i.v. continuous infusions of saline (0.02 ml/min), or 5-CT (0.1 μg/kg min). Ten min later, a D-R curve was elicited again during the infusion of the above compounds.

#### Other procedures applying to protocols I and II

The doses of all compounds used in the pharmacological tests of the present study have previously been reported^16,17,22^. The 5-HT receptor agonists and saline were continuously infused at a rate of 0.02 ml/min by a WPI model sp100i pump (World Precision Instruments Inc., Sarasota, FL, U.S.A.). The intervals between the different stimulation frequencies or noradrenaline doses depended on the duration of the tachycardic responses (5–10 min), as we waited until heart rate had returned to baseline values.

### Immunohistochemistry analysis of 5-HT receptors

For this purpose, as previously pointed out, 5 normoglycaemic and 5 diabetic rats were used; these animals were sacrificed with an overdose of sodium pentobarbital (180 mg/kg; i.p.). Then, the upper mediastinal block was extracted by careful dissection, together with the content of the clavicular space, which was fixed overnight by immersion in 4% paraformaldehyde in phosphate buffer (0.1 M, pH = 7.4) for subsequent inclusion in paraffin and obtaining serial sections of 7 microns thick. For immunohistochemistry, the slides were previously deparaffinized and rehydrated. Afterwards, they were washed three times in TBS (Trizma-HCl buffered saline 0.05 M, pH = 7.4, plus 0.9% NaCl, used as the solution for washes and dilutions). The non-specific reactions were blocked by incubation in normal swine serum (Dako, diluted 1:30 in TBS) for 30 min at room temperature. Then, the sections were incubated in the primary rabbit polyclonal antibodies against: (i) 5-HT_1B_ receptor internal region (ABIN3180882, Online antibodies, diluted 1:200 in TBS), (ii) 5-HT_1D_ receptor (ABIN3181713, Online antibodies, diluted 1:200 in TBS), and (iii) 5-HT_5A_ receptor (ABIN1386672, Online antibodies, diluted 1:300 in TBS) overnight at 4° C in a humidity chamber. After washing, the slides were incubated for 2 h with goat anti-rabbit IgG Alexa Fluor 488 (Abcam, diluted at 1:600 in TBS). Afterwards, the sections were washed again with TBS followed by immersion in Mayer's haematoxylin to visualize the nuclei and identify anatomical structures. Slides were mounted with fluoromount aqueous mounting medium (Sigma). The fluorescence visualization in slides was obtained by a confocal microscope TCSSP2 (Leica). Z-stack of 5 × 1 μm sections was captured from the stellate ganglion. From 40 × high-resolution micrographs, fluorescence intensity was determined as arbitrary units of grey level with ImageJ 1.49 free software (developed by the National Institutes of Health, U.S.A, and downloaded from https://imagej.nih.gov/ij).

### Data analysis and statistical procedures

All data in the text, tables and figures are presented as the mean ± SEM. Initially, the difference between the changes in blood glucose and gain of body weight in normoglycaemic and diabetic animals was evaluated using Mann–Whitney U test. The peak changes in heart rate by cardiac sympathetic stimulation or exogenous noradrenaline in the saline- and 5-HT receptor agonists-infused rats were determined. The difference in the values of heart rate and diastolic blood pressure within one (sub)group of rats before and during the infusions of saline or the 5-HT receptor agonists, as well as the immunohistochemistry data, were evaluated with paired Student’s t-test. Moreover, the difference between the changes in heart rate within one subgroup of animals was evaluated with the Student–Newman–Keuls test, once a two-way repeated measures ANOVA (randomized block design) had revealed that the samples represented different populations. Additionally, the difference between measured baseline haemodynamic variables in diabetic and normoglycaemic pithed rats was evaluated using a one-way ANOVA, followed by the Student–Newman–Keuls post hoc test. Statistical significance was accepted at *P* < 0.05 (two-tailed).

### Pharmacological compounds

Apart from the anaesthetic (sodium pentobarbital), the drugs used in the present study (and their corresponding source) were as follows: streptozotocin (STZ), citric acid, sodium citrate, gallamine triethiodide, desipramine hydrochloride, noradrenaline hydrochloride, 5-HT creatinine sulphate and SB 699551 (N-[2-(dimethylamino)ethyl]-N-[[4′-[[(2-phenylethyl)amino]methyl][1,1′-biphenyl]-4-yl]methyl]-cyclopentanepropanamide dihydrochloride) were obtained from Sigma Chemical Co. (St. Louis, MO, U.S.A.); 5-carboxamidotryptamine (5-CT) maleate was obtained from GlaxoSmithKline (Stevenage, Hertfordshire, U.K.); indorenate (5-methoxytryptamine-β-methylcarboxylate hydrochloride) was a gift from Prof. Enrique Hong, Cinvestav-IPN (Mexico City, Mexico); CP 93,129 (3-(1,2,5,6-tetrahydropyrid-4-yl)pyrrolo[3,2-b]pyrid-5-one was acquired from Pfizer Inc. (Groton, CT, U.S.A.); LY344864 (N-[3-(dimethylamino)-2,3,4,9-tetrahydro-1H-carbazol-6-yl]-4-fluorobenzamide) was obtained from Eli Lilly & Co. (Indianapolis, IN, U.S.A.); and PNU 142633 [(S)-(−)-3,4-dihydro-1-[2-[4-aminocarbonyl)phenyl]-1-piperazinyl]ethyl]-N-methyl-1H-2-benzopyran-6-carboxamide] was a gift from Dr. R.B. McCall, Pharmacia & Upjohn (Kalamazoo, MI, U.S.A.). Physiological saline was utilized as dissolvent for all compounds, except for STZ, which was dissolved in citrate buffer (0.1 M; pH 4.5)^22,28,29^; these vehicles had no effect on the basal heart rate or basal diastolic blood pressure (not shown). Except for noradrenaline, desipramine, and gallamine (whose doses represent the appropriate salts), the doses described in the manuscript represent the free base of all compounds^22,28,29^.

### Ethics approval

All the animal procedures and protocols of the present investigation were reviewed and approved by our Institutional Ethics Committee on the use animals in scientific experiments (CICUAL Cinvestav; protocol number 507-12) and followed the regulations established by the Mexican Official Norm (NOM-062-ZOO-1999), in accordance with: (i) the guide for the Care and Use of Laboratory Animals in the U.S.A.; and (ii) the ARRIVE guidelines for reporting experiments involving animals^59^.

## Data Availability

The data that support the findings of this study are available from the corresponding author upon reasonable request.
